# Important Crop Pollinators Respond Less Negatively to Anthropogenic Land Use Than Other Animals

**DOI:** 10.1002/ece3.70486

**Published:** 2024-10-30

**Authors:** Jessica J. Williams, Tim Newbold, Joseph Millard, Vivienne P. Groner, Richard G. Pearson

**Affiliations:** ^1^ Centre for Biodiversity and Environment Research, Department of Genetics, Evolution and Environment University College London London UK; ^2^ Department of Life Sciences Natural History Museum London UK; ^3^ Department of Life Sciences Imperial College London, Silwood Park Campus Berkshire UK

**Keywords:** cropland, ecosystem services, land‐use change, pollination, PREDICTS

## Abstract

Animal‐mediated pollination is a key ecosystem service required to some extent by almost three‐quarters of the leading human food crops in global food production. Anthropogenic pressures such as habitat loss and land‐use intensification are causing shifts in ecological community composition, potentially resulting in declines in pollination services and impacting crop production. Previous research has often overlooked interspecific differences in pollination contribution, yet such differences mean that biodiversity declines will not necessarily negatively impact pollination. Here, we use a novel species‐level ecosystem service contribution matrix along with mixed‐effects models to explore how groups of terrestrial species who contribute differently to crop pollination respond globally to land‐use type, land‐use intensity, and availability of natural habitats in the surrounding landscape. We find that the species whose contribution to crop pollination is higher generally respond less negatively (and in some cases positively) to human disturbance of land, compared to species that contribute less or not at all to pollination. This result may be due to these high‐contribution species being less sensitive to anthropogenic land conversions, which has led humans to being more reliant on them for crop pollination. However, it also suggests that there is potential for crop pollination to be resilient in the face of anthropogenic land conversions. With such a high proportion of food crops requiring animal‐mediated pollination to some extent, understanding how anthropogenic landscapes impact ecological communities and the consequences for pollination is critical for ensuring food security.

## Graphical abstract

With a high proportion of crops requiring animal‐mediated pollination to some extent, understanding how anthropogenic landscapes impact communities and pollination provision is critical for ensuring food security. We find that the species most important for crop pollination are responding less negatively (and sometimes positively) to human disturbance of land compared to other animals. This suggests there is potential for crop pollination to be resilient in the face of anthropogenic land conversions.

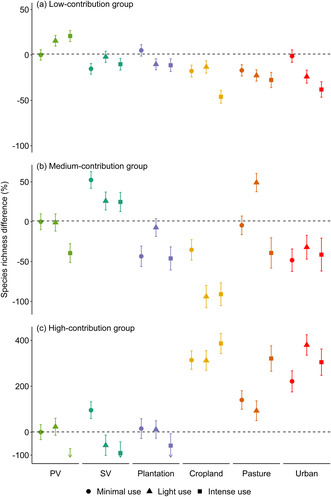

## 1 Introduction

Anthropogenic pressures are causing shifts in the composition of ecological assemblages (Millennium Ecosystem Assessment 2005; Newbold et al. 2015; Walther et al. 2002). This can lead to declines in ecosystem functioning and impact the benefits that nature provides to people (Cardinale et al. 2012; Mace, Norris, and Fitter 2012). These benefits are often referred to as ecosystem services and they underpin human well‐being (WWF 2018; Biggs et al. 2012). One such example is animal‐mediated pollination. This service is required to some extent by around three‐quarters of human food crop species (Klein et al. 2007), benefitting the global economy by an estimated US$235‐577 billion (2015 US$) through increasing worldwide annual crop output (Lautenbach et al. 2012; Potts et al. 2016). However, anthropogenic pressures such as habitat loss and land‐use intensification can impact abundance and richness of pollinators across ecological communities (Biesmeijer et al. 2006; Dicks et al. 2021; Potts et al. 2010; Soroye, Newbold, and Kerr 2020). These shifts in ecological assemblages may result in declines in pollination provision, negatively impacting human well‐being. For instance, it is estimated that 5%–8% of global crop production (by mass) would be lost without animal‐mediated pollination, imposing dietary changes and requiring the transformation of more land to agriculture to keep up with food demands (particularly in the Global South (Aizen et al. 2009)). Furthermore, the crops that are dependent to some degree on animal‐mediated pollination (such as fruits, nuts, and vegetables) are important for providing key nutrients and variety in our diets (Smith et al. 2015). Understanding the impact of anthropogenic pressures on pollinator assemblages is therefore crucial if we are to sustain current and future societal needs (Biggs et al. 2012; Potts et al. 2016; Dicks et al. 2021).

Land use (e.g., natural habitats versus agricultural and urban areas), land‐use intensity (e.g., level of chemical input or resource extraction), and landscape composition (the proportion of natural or human‐altered land uses surrounding a site) are known to impact ecological assemblages, particularly pollinators, with effects differing between species (Newbold et al. 2015; Millard et al. 2021; Ganuza et al. 2022; Kennedy et al. 2013; Steffan‐Dewenter and Westphal 2008). For example, pollinator diversity has been found to be lower in human‐altered land uses, particularly those used more intensively by humans, compared to more natural habitats (Millard et al. 2021; Ganuza et al. 2022). However, species richness of pollinators in areas of low or intermediate disturbance can be higher than in natural habitats (Millard et al. 2021). At the landscape level, landscape composition is an important factor influencing pollinator species richness and abundance (Ganuza et al. 2022; Cariveau et al. 2013), with biodiversity harboured in habitats adjacent to cropland known to influence pollinator‐dependent crop production (Tscharntke et al. 2022; Kremen, Williams, and Thorp 2002; Carvalheiro et al. 2011). Previous research has found that landscape composition influences pollinators to a greater degree than landscape configuration (the distribution, abundance, and size of different land‐use types surrounding a site (Ganuza et al. 2022; Kennedy et al. 2013; Jauker et al. 2009)). For instance, species richness of moths and flies has been found to be higher in sites surrounded by a lower proportion of agriculture and grassland (Ganuza et al. 2022). For bees, species richness has been found to be higher in sites surrounded by greater proportions of urban areas (at the expense of forest, grassland, or agriculture (Ganuza et al. 2022)). However, not all taxa respond in the same way, with the influence of the proportion of surrounding land in agricultural production differing between bee species (Cariveau et al. 2013). These differences between taxa in responses to anthropogenic land uses and landscape composition cause species turnover along environmental gradients (Quintero, Morales, and Aizen 2010; Newbold et al. 2016; Rabello et al. 2021; Almeida‐Maués et al. 2022).

As well as responding differently to land‐use characteristics, species do not contribute equally to pollination (Herrera and Pellmyr 2002; Rader et al. 2011). For example, it has previously been suggested that, in general, the more abundant (dominant) species are more important for pollination (Winfree et al. 2015). The difference between species is also in part due to crops differing in their dependence on animal‐mediated pollination (Klein et al. 2007). For example, animal‐mediated pollination is essential for watermelon (*Citrullus lanatus*) and kiwi (*Actinidia deliciosa*), whereas there is little loss of production without animal visitors for tomato (*Lycopersicon esculentum*) and papaya (*Carica papaya*) (Klein et al. 2007). Consequently, the loss of those species that pollinate crops for which animal‐mediated pollination is essential will pose a larger risk to crop production compared to species known to pollinate crops that are not dependent on this form of pollination. For instance, whilst bees are often highlighted as the most important crop pollinators (Rader et al. 2016; Jauker et al. 2012), the loss of some species, such as *Peponapis pruinosa* that pollinate watermelon, may pose higher risks to crop production than the loss of species such as *Amegilla chlorocyanea*, which pollinate tomatoes (Klein et al. 2007). This also means that, for some crops, maintaining sufficient pollination levels may rely on species that are rare or declining (Genung et al. 2023).

Past research has found that despite widespread declines in British bee species, dominant crop pollinators increased in occupancy from 1980 to 2013 (Powney et al. 2019). As such, changes in the composition of ecological assemblages may not always have negative consequences for pollination services. There may be functional redundancy within an assemblage (Nyström 2006), or species moving into an area may perform similar functions to those becoming locally extinct, or the assemblage may gain pollinators that contribute more to pollination than those lost. On the other hand, if an ecological assemblage loses its most important pollinators, and species entering the area are not pollinators, or are poor pollinators for the community of plants in that location, there may be pollination shortages, leading to reduced or unstable yields for crops that have high dependence on animal‐mediated pollination. This leads to the following question: are species with different contributions to pollination responding differently to anthropogenic land uses, and what impact may this have on animal‐mediated pollination following shifts in the composition of ecological assemblages driven by land‐use change?

Previous work to understand the spatial responses of pollinating species to land use has tended to group all pollinator species together (thus not considering differences in pollination contribution (Millard et al. 2021)), focus on certain taxa (Cariveau et al. 2013; Oliver et al. 2015; Winfree and Kremen 2009; Lázaro et al. 2016), or classify any flower visitor as a pollinator (Ganuza et al. 2022; Cusser, Neff, and Jha 2018). Overlooking species' different contributions to pollination has limited the ability of past research to understand how shifts in ecological assemblages within anthropogenic landscapes may be impacting pollination. To our knowledge, the impact of land use, land‐use intensity, and natural habitat availability on species that differ in their pollination contribution has yet to be assessed. Here, we use a novel matrix approach to quantify the contribution of different species within an ecological assemblage to crop pollination provision. Specifically, we assess the contribution of a species to crop pollination by considering both the importance of a species for pollination and the uncertainty underlying the evidence for this importance. We investigate whether species with different contributions to pollination vary in their responses to land use and land‐use intensity as well as the availability of natural habitats in the surrounding landscape. As such, we make a first step at accounting for interspecific differences in pollination provision when looking at the impacts of anthropogenic landscapes on animal‐mediated pollination services globally. As a result of the research highlighted above on species' different responses to anthropogenic landscapes (Ganuza et al. 2022; Cariveau et al. 2013) and the increase in dominant crop pollinators occupancy over the last few decades (Powney et al. 2019), we expected that groups of species that differ in their contribution to crop pollination will not respond uniformly to landscape features. As species that pollinate crops are more likely to be present in agricultural areas, we predicted that species whose contribution to crop pollination is higher may be less negatively impacted by human‐altered landscapes, although the expected magnitude and direction of effects of land use on groups of species contributing differently to agricultural pollination remains unclear. Further, it is unknown how land‐use intensity and availability of natural habitats in the landscape will impact pollinators with different levels of contribution to agriculture. Together, our results will help to understand the potential resilience of pollination to future species turnover driven by human disturbance of land.

## 2 Methods

### 2.1 Ecological Assemblage and Land‐Use Type Data

We acquired occurrence and abundance data for terrestrial animal species (including likely pollinators and species not likely to provide pollination services) from the PREDICTS (Projecting Responses of Ecological Diversity in Changing Terrestrial Systems) Project database (Hudson et al. 2016, 2017). This database contains data from studies across the globe that have made spatial comparisons of ecological assemblages across different land‐use types and intensities (Hudson et al. 2014). It is hierarchically structured, whereby the database contains data from published *sources* that each contain data from one or more *studies* (split by sampling method or split due to covering large geographic areas, such as multiple countries), which may themselves be split into *spatial blocks* (for spatially blocked designs), and then into *sites* (where sampling of the ecological assemblage occurs). For each site, the database contains records of species' abundance, occurrence, or overall species richness of sampled taxa (for more information, see (Hudson et al. 2017; Hudson et al. 2014)). We selected only sites with geographic coordinates (so we could obtain landscape composition data) and land‐use type and intensity classifications. We removed data for species whose binomial name was not recorded, was uncertain, or was incomplete. We also used the MergeSites function within the ‘predictsFunctions’ package (Newbold 2018a) to merge sites that had the same coordinates, and that came from the same original study, used the same sampling methods on the same sampling dates, and had the same land‐use type and intensity. We included all sites that met the above criteria (i.e., including studies focusing on pollinator species as well as those that did not sample any likely pollinators so that we could compare across species groups). Overall, we obtained occurrence data for 11,861 assemblages, containing 13,701 species (5 Adenophorea, 334 Amphibia, 1068 Arachnida, 2970 Aves, 24 Chilopoda, 26 Clitellata, 28 Diplopoda, 103 Entognatha, 269 Gastropoda, 8029 Insecta, 20 Malacostraca, 519 Mammalia, 302 Reptilia, 2 Secernentea, 2 Udeonychophora). Of these, 13,051 species had abundance data.

Within the PREDICTS database, each site containing an assemblage is assigned a land‐use type (Table S1) (Hudson et al. 2014). We considered six different land‐use types: (1) primary vegetation—natural vegetation with no evidence of previous destruction (by humans or extreme natural events), (2) secondary vegetation—vegetation recovering from destruction, (3) plantation forest—agricultural land used for cultivating woody crops, (4) cropland—agricultural land used to cultivate herbaceous crops, including animal fodder, (5) pasture—agricultural land used for livestock grazing, and (6) urban areas—areas of human habitation and buildings. In the following, we refer to human‐altered land uses as including plantation forests, croplands, pastures, and urban areas. Each site is also assigned a use intensity: minimal, light, or intense. The criteria used to classify use intensity is specific to each land‐use type and is based on factors such as chemical input, stock density, crop rotation, irrigation, logging and bushmeat extraction (see Table S1) (Hudson et al. 2014). Therefore, we combined land‐use type and land‐use intensity categories into a single axis of disturbance with 18 categories (e.g., light use plantation forest, intense use cropland, minimal use pasture; following (Millard et al. 2021; De Palma et al. 2016)).

### 2.2 Landscape composition

To account for landscape composition, we calculated the percentage of semi‐natural habitat (SNH) surrounding each site using the 2005 global land‐cover map from the European Space Agency Climate Change Initiative (ESA CCI (ESA Land Cover CCI Project Team and Defourny 2019)). This land‐cover map has a spatial resolution of 300 m and contains 37 land‐cover categories (Table S2) (Defourny et al. 2017). We chose the year 2005 as this was the mean of the midpoint year of sampling of the sites in our dataset. We grouped land‐cover categories to calculate the percentage of surrounding SNH within a 1‐km radius of each site using the method of Williams et al. (2022). SNH included forest, grassland, wetland and shrubland, and excluded all other categories (e.g., agriculture, urban, or bare areas (Williams et al. 2022)). When calculating percentage of SNH we accounted for the maximum percentage cover detailed within each of the ESA's land‐cover categories (Williams et al. 2022). For example, the category ‘Tree cover, needleleaved, evergreen, closed (> 40%)’ could cover 100% of the 300 × 300‐m area, whereas the category ‘Tree cover, needleleaved, evergreen, open (15%–40%)’ could cover a maximum of 40% of the 300 × 300‐m area. We used a radius of 1 km because this has previously been chosen to approximate the dispersal distance of a wide range of taxa (Ganuza et al. 2022; Williams et al. 2022; Le Provost et al. 2020). We also checked the sensitivity of our results to including the percentage of semi‐natural habitat within a 10‐ and 50‐km radius of each site, and to using land‐cover data from a different year (2002 and 2008, which are the lower and upper quartiles of the midpoint year of sites sampling years).

### 2.3 Species‐Level Ecosystem Service Contribution Matrix

**FIGURE 1:**
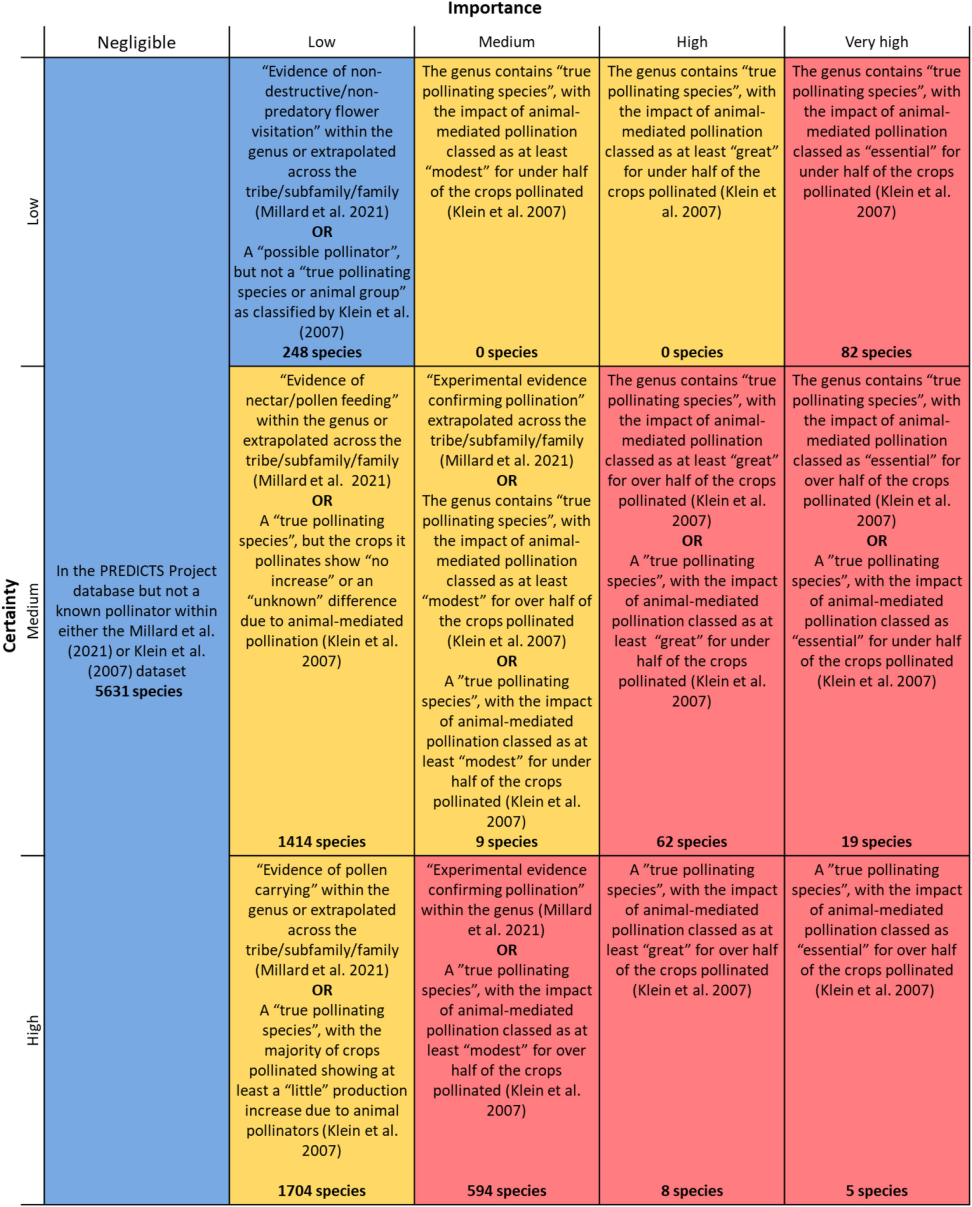
The species‐level ecosystem service contribution matrix for pollination. ‘Importance’ refers to the importance of a species for pollination provision, and ‘Certainty’ refers to the confidence underlying the evidence for the species' importance. Each cell includes the definitions used to classify each species, and the number of species in our analyses with this classification (in bold). The colour of the cell gives an indication of the contribution of those species to pollination provision: Blue = low contribution, yellow = medium contribution, red = high contribution. We only included species from Orders found across all contribution groups (low, medium, and high). A ‘true pollinating species or animal group’ is defined by Klein et al. (2007) as “species for which at least 80% of their single flower visits result in a fruit, or species that lead to higher fruit and seed quality and quantity when caged or abundant in natural communities in contrast to fruit and seed quality and quantity when flowers are protected from all visitors”. A ‘possible pollinator’ within the Klein et al. (2007) database is a floral visitor. If a species could have fit into two different classifications, we assigned the species the higher risk classification (e.g., if there was evidence confirming pollination at the genus level within Millard et al. (2021), but the species was classed as a “true pollinator” with the impact of animal‐mediated pollination classed as “great” for over half of the crops it pollinated within Klein et al. (2007), the species was assigned with a contribution classification of High Importance, High Certainty). See Table S5 for the list of species names in each contribution group.

To incorporate interspecific differences in contribution to pollination and assess how differences in ecological assemblages across land uses may impact potential pollination provision, we developed a novel *species‐level ecosystem service contribution matrix*. The species‐level ecosystem service contribution matrix combines (1) the ‘Importance’ of a species for the provision of a particular ecosystem service (pollination of crops in the case of this study) and (2) the ‘Certainty’ underlying the evidence for this importance (which can depend on the evidence source—e.g., published data vs. expert opinion—or the taxonomic level that we have data for—e.g., genus or species), to produce a set of contribution classifications (Figure 1). These classifications give an indication of a species' contribution to the provision of the ecosystem service, and can be used as a proxy for the impact that the loss of that species (either completely or thorough reduced abundance) may have on service provision. The species‐level ecosystem service contribution matrix is similar to ecosystem service matrices, which have been used to guide management decisions by identifying the importance of habitats (and occasionally species) for ecosystem service provision and the certainty underlying this importance (Burkhard et al. 2009; Campagne et al. 2017; Geange et al. 2019; Kokkoris et al. 2019; Potts et al. 2014). However, to our knowledge, the two measures (importance and certainty), within species‐level ecosystem service matrices (which have so far been restricted to marine species (Potts et al. 2014; Burdon et al. 2017)), have not previously been combined to give an indication of contribution, or used to assess how responses to anthropogenic pressures may vary across species that differ in their service contribution. Overall, the species‐level ecosystem service contribution matrix provides a relatively simple approach to explore the responses of species with differing ecosystem service contributions to environmental change.

The species‐level ecosystem service contribution matrix also offers two key advantages for our study. First, we are able to incorporate interspecific differences in contribution to crop pollination, which is vital if we are to understand how shifts in ecological assemblages due to environmental changes may impact pollination. Second, it provides the ability to account for the certainty of evidence underlying pollination contribution, and thus account for the different ways that previous studies have classified pollinators (e.g., all species that visit flowers (Ganuza et al. 2022), versus species for which there is evidence of pollen carrying (Rader et al. 2011), versus experimental confirmation or quantification of animal‐mediated pollination (Liu et al. 2020; Ollerton and Liede 1997)).

We acquired data on species' provision of pollination services from Millard et al. (2021) and Klein et al. (2007). The pollinator dataset constructed by Millard et al. (2021) contains a list of genera that are pollinators (for any flowering plant) and was produced using an automatic text‐analysis method followed by manual inspection. Millard et al. (2021) also carried out searches within higher‐level taxonomic groups to extrapolate across genera if there was sufficient evidence. Further, this pollinator dataset was assessed by a set of experts who removed any taxa that were highly unlikely to be pollinators (Millard et al. 2021). The dataset constructed by Klein et al. (2007) contains 107 food crops important on the world market (that are directly consumed by people), their level of dependence on animal‐mediated pollination, and their likely pollinators. Two key features of these datasets enabled us to create the species‐level ecosystem service contribution matrix (see Figure 1). First, they include a level of uncertainty in their classifications: Millard et al. (2021) includes a four‐level confidence score, ranging from the highest confidence where there was experimental evidence confirming pollination for at least one species in the genus, to the lowest confidence where only non‐destructive/non‐predatory flower visitation had been observed; and Klein et al. (2007) differentiates between ‘true pollinators/primary pollinators’ (defined as ‘species for which at least 80% of their single flower visits results in a fruit (Klein, Steffan‐Dewenter, and Tscharntke 2003a; Klein, Steffan‐Dewenter, and Tscharntke 2003b) or species that improve the fruit and seed quality and quantity when abundant as compared with the level when all flower visitors are excluded’) and possible pollinators (i.e., floral visitors). Second, Klein et al. (2007) completed an extensive literature review in order to include detail on how important animal pollination was for the production of each crop in the dataset (based on the reduction in production without flower visitors), which meant we could assess the importance of pollinator species. For example, species that pollinate crops for which animal‐mediated pollination was more important (e.g., if animal‐mediated pollination was classed as great or essential for crop production) were considered to be of higher importance compared to species that pollinated crops for which animal‐mediated pollination had little impact on production (Figure 1). For further description of these databases, see the Supporting Information.

**FIGURE 2:**
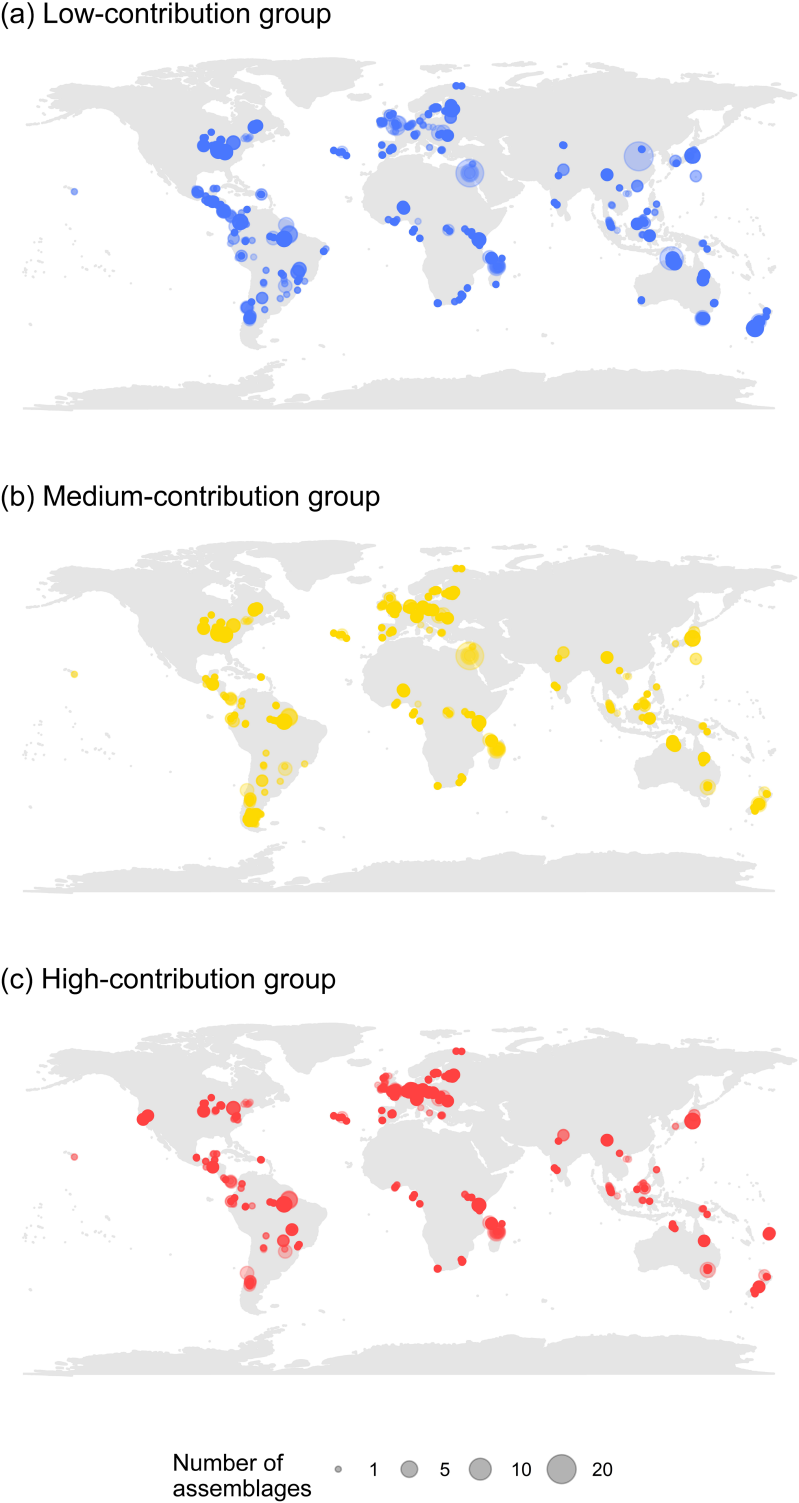
The spatial distribution of assemblages within the PREDICTS Project database that included species within (a) low‐, (b) medium‐, and/or (c) high‐contribution groups from the Orders of Apodiformes, Chiroptera, Coleoptera, Didelphimorphia, Diptera, Hymenoptera, Lepidoptera, Passeriformes, or Squamata. Contribution group indicates the contribution of the species towards the provision of crop pollination (see Figure 1).

We used the data in Millard et al. (2021) and Klein et al. (2007) to develop a species‐level ecosystem service contribution matrix for pollination, giving each cell within the matrix a definition based on the information included in the two datasets (Figure 1). To assign a species' level of importance, we looked at the relationship between the species (or genera) and flowers/crops (e.g., whether the species is an unlikely pollinator vs. evidence of pollination) and, for pollinating species and genera listed in Klein et al. (2007), how dependent the crops they pollinate are on animal‐mediated pollination. The level of certainty was assigned by looking at (1) the taxonomic resolution of the evidence (we assigned a higher certainty classification to a species if the species was listed as a pollinator rather than the genus; if the genus was listed as a pollinating genera, we assumed all species within the genera were pollinators; Figure 1), (2) for flower visitors, the evidence suggesting that the species *may* pollinate (e.g., evidence of non‐predatory flower visitor vs. evidence of pollen feeding), (3) whether the evidence was available for crops specifically or flowers more generally, and (4) for known crop pollinators, the proportion of crops that they pollinated for which animal‐mediated pollination had an essential/great/modest/little impact on crop production. Note that from Klein et al. (2007) we only used information on named genera or species, we excluded data on broader/less precise categories, such as ‘flies’, ‘bats’, or ‘ants’.

Using the species‐level ecosystem service contribution matrix (Figure 1), we assigned each species in our dataset a contribution classification. Following the ‘traffic light’ format of a risk matrix (Jordan, Mitterhofer, and Jørgensen 2018; Li, Bao, and Wu 2018), we categorised the cells in the matrix into three groups according to the contribution towards pollination provision (indicated by colour in Figure 1): (1) low contribution (blue), (2) medium contribution (yellow), and (3) high contribution (red). Across the contribution groups, there were bird, insect, mammal, and reptile species in all groups (Tables S3, S4), whereas species from the other Classes in our dataset (Adenophorea, Amphibia, Arachnida, Chilopoda, Clitellata, Diplopoda, Entognatha, Gastropoda, Malacostraca, Secernentea and Udeonychophora) only occurred in the low‐contribution group. Due to this imbalance across contribution groups, we dropped any Orders that did not include species in all contribution groups. Our final dataset included 9776 species across the following nine Orders: Apodiformes, Chiroptera, Coleoptera, Didelphimorphia, Diptera, Hymenoptera, Lepidoptera, Passeriformes, and Squamata (Appendix A, Table A1; Tables S5, S6). For each contribution group, the land‐use‐use‐intensity categories contained species from between 70—1409 assemblages (Appendix A, Table A1). The exceptions included intense use pasture, which contained species within the low‐contribution group from 52 assemblages (of which 46 assemblages had abundance data) and intense use urban sites, where there were species within the medium‐ and high‐contribution groups from 39 assemblages (see Appendix A, Table A1).

We assessed the spatial coverage of data for each contribution group; our dataset contained assemblage data across 9492 sites (7472 contained species with low‐contribution group, 6329 contained medium‐contribution group species, and 5643 contained high‐contribution species; Figure 2; also see Tables S7, S8), and these sites ranged in the percentage of SNH from 0% to 100% (Table S9).

### 2.4 Statistical Analyses

To explore how groups of terrestrial species with different contribution levels to pollination respond to anthropogenic landscapes, we analysed how species richness and total abundance of the three contribution groups from the species‐level ecosystem service contribution matrix were impacted by land‐use type, use intensity, and landscape composition.

We used a generalised linear mixed‐effects model with a Poisson error distribution to explore the impact of land‐use type and intensity on species richness (the number of uniquely named species within an assemblage). We used backwards stepwise variable selection, which uses maximum likelihood estimation to select terms and likelihood‐ratio tests to compare the fit of different models (Zuur et al. 2009) (Appendix A, Table A2). The fixed effects included in the backwards stepwise variable selection were contribution group (categorical variable) and the land‐use‐use‐intensity variable (categorical variable), and the interaction between them. We did not include percentage of surrounding SNH, as our model would not converge with this addition. Following previous studies using the PREDICTS Project database (Newbold et al. 2015; Millard et al. 2021), we included three random intercept terms: (1) study identity, to account for variation across studies in methods or measures used, (2) spatial block, to account for the spatial structuring of sites where assemblages were sampled, and (3) site identity, to control for overdispersion within the estimates of species richness (Rigby, Stasinopoulos, and Akantziliotou 2008). We checked for overdispersion in these models using the R package ‘StatisticalModels’ (Newbold 2018b), and applied a quasi‐likelihood analysis to adjust for overdispersion.

We used a linear mixed‐effects model to investigate the impact of contribution group, land‐use type, land‐use intensity, and percentage of surrounding SNH on total abundance (the sum of the relative abundances of all species sampled within an assemblage). We log_e_(*x* + 1) transformed the total abundance values to normalise the model residuals (Millard et al. 2021). Again, we used backwards stepwise variable selection to select main effects and interactions. Into the backwards stepwise variable selection, as potential explanatory variables, we included contribution group, land‐use‐use‐intensity (categorical variables), the percentage of surrounding SNH within a 1‐km radius (continuous variable), and the 2‐ and 3‐way interactions between these variables as fixed effects. We included study identity and spatial block as random intercept terms. For both the species richness and total abundance models, all variables included in the backwards stepwise variable selection were included in the best‐fit models (Appendix A, Table A2).

### 2.5 Sensitivity Tests

We carried out a range of sensitivity tests to ensure our models were robust. First, we calculated Chao1‐estimated species richness to check whether our results differed when accounting for incomplete sampling (Chao, Chazdon, and Shen 2005). Second, we used a zero‐inflated negative binomial mixed model to investigate the impact of landscape features on total abundance, accounting for the large number of abundance counts that are 0 (*n* = 4198). We also ran an ‘overall’ species richness and total abundance model (i.e., by removing the contribution grouping and including the total number of species or abundance of individuals at a site in the models, respectively) and used Moran's I tests to check for spatial autocorrelation in the residuals of each individual study. Further, across all assemblages in the dataset, total abundance measures ranged from 0 to over 350,000, due to some assemblages including very large groups/flocks of species such as African fig flies (*Zaprionus indianus*). Most assemblages (*n* = 18,038) had a total abundance of less than 5000. To ensure that the extreme abundance measures did not impact our results, we reran the total abundance model excluding outliers. Outliers were defined as:

(1) $$O>Q_{3}+1.5^{*}\mathrm{IQR}$$

where O denotes outliers, and Q_3_ and IQR are the third quartile and the interquartile range of total abundance measures, respectively. We also ran leave‐one‐out cross‐validation tests where studies were dropped one by one, and each model rerun to ensure there were no overly influential studies in our dataset. Additionally, we tested the robustness of our results to basing contribution groupings on importance alone, and not incorporating the certainty scale. To do this, we used the contribution groupings indicated along the high certainty row for each importance grouping (negligible, low, medium, high, very high), so that a species' importance for pollination was not down‐weighted by its level of certainty. Using these new contribution groupings, we then ran the same models described above.

All analyses were completed in R 4.1.0 (R Core Team 2021) using the packages ‘dplyr’ v.1.0.8 (Wickham et al. 2022), ‘lme4’ v.1.1.28 (Bates et al. 2015), ‘plyr’ v.1.8.6 (Wickham 2011), ‘predictsFunctions’ (Newbold 2018a), ‘StatisticalModels’ v.0.1 (Newbold 2018b), ‘Hmisc’ v.4.6.0 (Harrell Jr 2021), and ‘NBZIMM’ (Zhang and Yi 2020). The extraction of the percentage of surrounding SNH was completed in ArcGIS 10.4 (ESRI 2015).

## 3 Results

**FIGURE 3:**
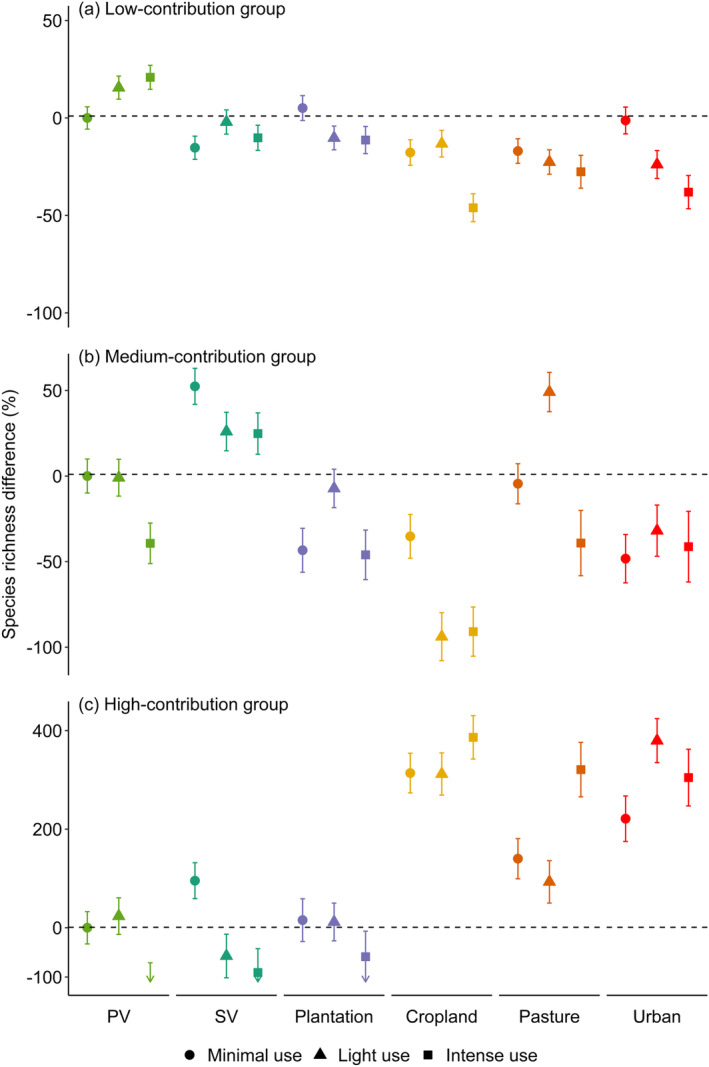
The difference in species richness in assemblages across different land‐use types and land‐use intensities, relative to that in minimally used primary vegetation. Assemblages have been split into three groups: (a) species in the low‐contribution group; (b) species in the medium‐contribution group; and (c) species in the high‐contribution group. Colours represent land‐use type: Primary vegetation (PV; light green), secondary vegetation (SV; dark green), plantation (purple), cropland (yellow), pasture (orange), and urban (red). Error bars represent ±1 SE. The down arrows for the high‐contribution group in intense use primary and secondary vegetation and plantation represent predictions by the model that there may be no species from the high‐contribution group found in these land uses. Note the difference in y‐axis limits between the plots.

Species richness differed across our land‐use type and intensity variable and, importantly, this differed with contribution grouping (Figure 3; Appendix A, Table A3). Whereas species richness for the low‐ and medium‐contribution groups tended to be lower across most human‐altered land uses relative to that in minimally used primary vegetation, this was not observed in the high‐contribution group. Species richness in the high‐contribution group was higher in cropland, pasture, and urban sites relative to minimally used primary vegetation (Figure 3c, Table A3). Further, across the low‐contribution group, richness in human‐altered land uses generally declined with higher use intensity (Figure 3a). However, across the medium‐ and high‐contribution groups, the impact of land‐use intensity did not always follow this pattern within human‐altered land uses (Figure 3b,c).

**FIGURE 4:**
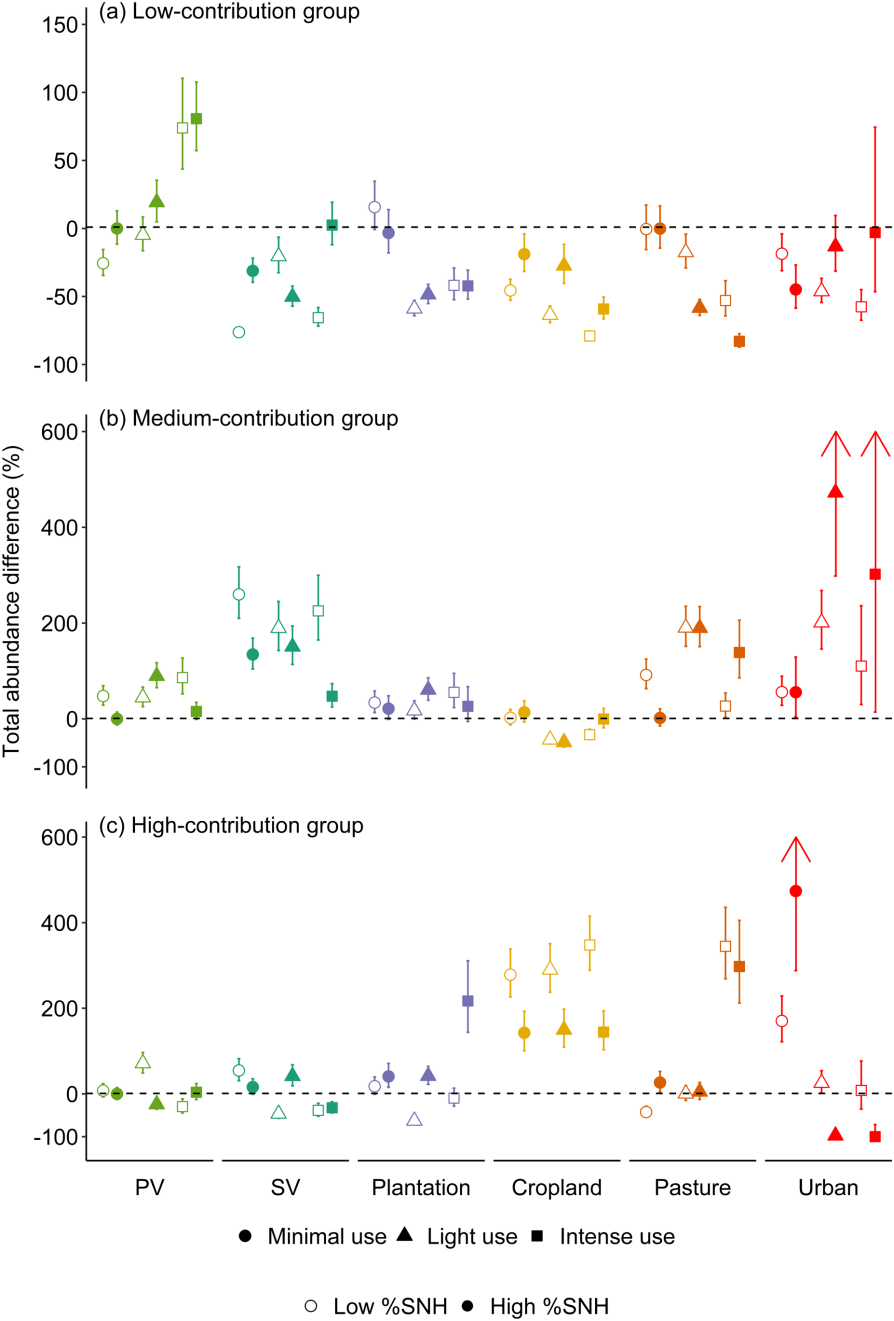
The difference in total abundance of species in assemblages across different land‐use types, land‐use intensities, and with different amounts of surrounding semi‐natural habitat (SNH), relative to assemblages in minimally used primary vegetation surrounded by a high percentage (91.4%) of SNH. Assemblages have been split into three groups: (a) species in the low‐contribution group; (b) species in the medium‐contribution group; and (c) species in the high‐contribution group. Open shapes represent those assemblages with a low percentage of surrounding SNH (37.5%) and filled shapes represent those assemblages with a high percentage of surrounding SNH (91.4%)—we chose to present these values as they were the 33rd and 66th percentile, respectively, across sampled sites. Colours represent land‐use type: Primary vegetation (PV; light green), secondary vegetation (SV; dark green), plantation (purple), cropland (yellow), pasture (orange), and urban (red). Error bars represent ±1 SE; the upper values of the error bars for light and intense use urban sites surrounded by a high percentage of SNH in plot (b) were 721% and 1253%, respectively; the upper values of the error bars for minimal use urban sites surrounded by a high percentage of SNH in plot (c) was 746%.

The interaction between land‐use‐use‐intensity categories and the percentage of surrounding SNH significantly impacted the total abundance, with direction of the effects again differing between contribution groups (*p* < 0.001, Figure 4; Appendix A, Table A4). For species in the low‐contribution group, compared to minimally used primary vegetation, abundances were lower within most human‐altered land uses, particularly those used more intensively (Figure 4a). However, for species in the high‐contribution group, in croplands (irrespective of SNH or use intensity), plantations surrounded by a high percentage of SNH, and intensively used pastures, we observed significantly higher total abundances compared to that in minimally used primary vegetation (Figure 4c). There was a similar trend towards higher abundances in plantations, pastures, and urban areas in the medium‐contribution group as well (Figure 4b). It is notable that across croplands, the total abundance of species in the high‐contribution group was higher for areas surrounded by a low compared to high percentage of SNH (Figure 4c), the opposite pattern to that observed for species in the low‐contribution group (Figure 4a).

### 3.1 Explanatory Power and Sensitivity Test Results

The models reported here had similar explanatory power to those presented in Millard et al. (2021), with a low percentage of variation explained by the fixed effects (1%–1.5%) and large percentage of variation by the random effects (70%–80%; Table S10). However, similar to Millard et al. (2021), our aim was to explore differences between species groups in the general trends of responses to the landscape features, not to predict biodiversity metrics for certain groups in specific locations, and the explanatory power of the models is acceptable for this purpose. The results of models using Chao1‐estimated species richness (Figure S1), or running zero‐inflated negative binomial mixed models (Figure S2), were similar to the results presented above, leading to the same overall conclusions with regard to the responses of different species groups. In the absence of spatial autocorrelation, we would expect by chance for the residuals associated with 5% of studies within the PREDICTS database to have a *p* value of < 0.05 when subjected to a Moran's I test. For the ‘overall’ species richness and total abundance model (i.e., the models without the contribution grouping variable), we found that the percentage of studies for which the residuals showed apparently significant spatial autocorrelation was 8.3 and 5.8, respectively (Table S11). On the whole, the results from the total abundance models run after removing outliers did not differ from those reported above (Figure S3). The exception was that, for medium‐contribution species, total abundance in intense use urban areas did not differ significantly from the total abundance in minimal use primary vegetation surrounded by a higher percentage of SNH. Following the leave‐one‐out cross‐validation tests, we looked at the effect of each study's removal on the estimated model coefficients and did not find any overly influential studies (Figures S4, S5). Basing contribution groupings on importance alone produced very similar results to those presented above (Figures S6, S7). Last, our finding that the abundance of species who contribute highly to pollination generally responded less negatively (and in some cases positively) to human disturbance of land, compared to groups of species that contribute less or not at all to pollination, was consistent when including the percentage of SNH within a larger (10‐ or 50‐km) radius (rather than within a 1‐km radius; Figures S8, S9), and when using land‐cover data from 2002 to 2008 (rather than 2005; Figures S10, S11).

## 4 Discussion

We find that species are not responding uniformly to human‐altered landscapes, with those species that have a higher contribution to crop pollination affected less negatively, and in some cases positively, compared to those that contribute less. Our results also demonstrate key differences in responses to land‐use intensity and landscape composition between species that vary in their pollination contribution. These findings, which highlight important differences between pollinators, are in line with past research that calls attention to the variation in responses of pollinating species (specifically, the occupancy of British bees and the difference between dominant crop pollinators and other bee species (Powney et al. 2019)). However, it is important to note that despite pollinators being present, their ability to pollinate may differ across land‐use types or be impacted by anthropogenic changes (e.g., the foraging ability of certain species can be impacted by change in pesticide use (Kremen et al. 2007)). Nonetheless, our research takes a step towards including interspecific differences and their underlying uncertainty when looking at the impacts of human‐altered landscapes on ecological assemblages and the ecosystem services they provide. Assessing the impacts of environmental changes on pollinators is vital for assessing future risk to food markets and livelihoods, mitigating negative impacts from anthropogenic pressures, and ensuring global food security (Potts et al. 2016, 2010).

The mechanisms underlying our results remain in question. Past research has shown that pollinator responses to croplands are more negative (much lower species richness and total abundance compared to that in natural habitats) in tropical compared to non‐tropical areas (Millard et al. 2021). Despite having a higher number of assemblages containing species within each contribution group in temperate areas (Table S8), our data still contained a high number of assemblages in tropical croplands (Table S7), and so we do not think that this is driving the less negative, and in some cases positive, response of the high‐contribution group to human‐altered land uses. However, there were fewer assemblages within tropical compared to temperate pastures, which could influence the result for this land‐use type. Unfortunately, there were not enough data to carry out separate analyses for temperate and tropical areas. As such, there may be differences in responses to temperate and tropical landscape composition that we are not able to identify in this study and would be of interest to examine if more data were available. An ecological mechanism underlying our results may be that the species we are currently relying on for crop pollination (those within the high‐contribution group) are, by necessity, more resilient to human‐altered land use (i.e., the species that are documented to be important pollinators are those that can survive in agricultural areas). Factors that could lead to species' being more resilient to human‐altered land uses include life‐history strategies (Albaladejo‐Robles, Böhm, and Newbold 2023), climatic tolerances (Williams, Bates, and Newbold 2020), and/or range sizes (Newbold et al. 2018). Further data on the potential contribution to crop pollination of species lost from human‐altered land uses could be used to explore (1) whether species within the high‐contribution group are those that are simply more resilient to land‐use change, and we are missing less resilient species that could contribute highly if they were present, or (2) if those species that are more resilient are also those that contribute more to crop pollination within ecological assemblages overall.

These findings have implications for the resilience of crop pollination under shifts in ecological assemblages following land‐use change. Our results suggest that due to high‐contribution species persisting in human‐altered land uses, if species' ability to pollinate crops is maintained across land uses, crop pollination may be resilient within these areas, particularly within croplands and pastures. We also observed that minimally used urban areas, which often include extensive green spaces (Hudson et al. 2014), appear to be benefitting important pollinator species. Past research that found positive effects of urban landscapes on pollinator species richness (Ganuza et al. 2022; Theodorou et al. 2020) suggested potential mechanisms including the larger number of flowering‐plant species in these environments and the diverse nesting resources available in urban areas (Ganuza et al. 2022; Baldock et al. 2015). It is important that urban areas maintain their pollinator communities, as it has been estimated that around 15%–20% of the world's total food supply originates from such areas (Armar‐Klemesu 2000). However, it should be noted that animal‐mediated pollination is not just important for food production, but also for the pollination of wild flowering plants (Genung et al. 2023). Moreover, a high species richness is important for pollination resilience, as diverse pollinator assemblages are required to maintain pollination rates over space and time (Lemanski, Williams, and Winfree 2022; Winfree et al. 2018). The species within the high‐contribution group are also being impacted by other anthropogenic pressures, such as climate change (Wyver et al. 2023). As such, despite our results suggesting that there is potential for crop pollination to be resilient across some land uses, this does not mean that the full pollination service (i.e., for wild and cultivated plants) provided by animals will be resilient against future anthropogenic land‐use changes.

We found that the effect of the interaction between land‐use type, use intensity and surrounding SNH differed between the contribution groups. Past work has highlighted the beneficial effects that low levels of human use intensity can have on maintaining or enhancing pollinator biodiversity, and the more negative effects of higher levels of human use (Millard et al. 2021). However, our results suggest that the impact of land‐use intensity may be more nuanced for species that contribute more highly to pollination and that it interacts with other landscape features (such as the availability of surrounding natural habitat). For example, in croplands and pastures, we found that species richness of the high‐contribution group was higher in intensely used sites compared to minimally and lightly used sites. We also observed that, as previous research has suggested (Ganuza et al. 2022; Williams et al. 2022), the effect of surrounding SNH is complex, differing across land‐use categories and contribution groups. Our results suggest that within croplands, areas surrounded by a lower percentage of SNH favour a higher total abundance of high‐contribution species compared to areas surrounded by a higher percentage of SNH. This could be due to the quality or type of surrounding SNH, which may be suboptimal for the species present in these areas (Bartual et al. 2019; Schoch et al. 2022), along with factors such as agri‐environmental schemes (Jauker et al. 2012), which have been designed to encourage and maintain pollinator populations within agricultural systems (Powney et al. 2019; Rural Payments Agency 2019). To disentangle the impact of land‐use intensity and SNH, further data are needed on site management and the quality, type, and intensity of the surrounding SNH as well as more data within tropical areas in order to examine geographical differences. Nevertheless, this highlights the importance of the combination of landscape features when looking at the impact of changes in land use across pollinating species.

The novel species‐level ecosystem service contribution matrix that we introduce here can be used to explore the impacts of an anthropogenic pressure on species that contribute differently to an ecosystem service. Previous work that grouped all pollinators together (Cariveau et al. 2013; Lázaro et al. 2016) overlooked these important interspecific differences in pollination provision. The advantages of the species‐level ecosystem service contribution matrix include that it accounts for such differences between species in their importance for an ecosystem service, as well as the uncertainty underlying the evidence for this importance (which is key when relying on multiple sources of evidence (Harwood 2000)). This method could be applied to any ecosystem service where there are sufficient data or expert knowledge to generate a contribution matrix and allocate species a contribution classification. Further, this approach is relatively simple, which is an important and appealing trait when trying to tackle complex questions, especially those of policy interest such as ways to enhance the resilience of ecosystem services (Biggs et al. 2012; Jordan, Mitterhofer, and Jørgensen 2018). Additionally, this approach is easy to update as and when new data become available.

There are, however, limitations to our approach. The importance of a species to pollination may change across space, there may be intraspecific differences (e.g., between the males and females of a species (Fuster and Traveset 2020)), and some species may have the potential to become important pollinators following environmental or ecological community changes. Unfortunately, we currently are not able to take these factors into account due to lack of available data. There may also be time‐lagged effects (i.e., a delay in the loss or gain of species following land‐use change), which are not captured in most space‐for‐time analyses (De Palma et al. 2018). However, for the assemblages in our dataset with data available, the median number of years since fragmentation or land‐use conversion was 13 (*n* = 1567), and past research has found that changes in populations and assemblages often occur rapidly following habitat change, with almost half of the changes occurring within 3 years (Daskalova et al. 2020). Consequently, we do not expect time‐lagged effects to be biasing our results. As mentioned above, one of the advantages of the species‐level ecosystem service contribution matrix is that it is a relatively simple approach and can be used to group species together into categories based on their contribution to an ecosystem service. However, currently this approach is not able to take into consideration synergistic or antagonistic interactions between species across an ecological assemblage when it comes to providing an ecosystem service (Frund et al. 2013), or to identify which specific species' roles may be functionally redundant. At a global scale, the data are not available for these factors to be included, but further localised field studies could work on gathering the data for future work. Furthermore, although our results suggest larger losses of species in the low‐ compared to high‐contribution group within human‐altered land uses, the loss of species within this low‐contribution group may lead to other ecosystem services declining, which may have detrimental impacts on crops. For example, a decline in predators of crop pests could lead to a decrease in crop production due to an increase in pest species (Tschumi et al. 2015).

Whilst there is still much to learn, our study takes steps towards accounting for interspecific differences in pollination provision when looking at the impact of anthropogenic land uses and landscapes on pollination. With such a high proportion of human food crops requiring animal‐mediated pollination to some extent (Klein et al. 2007), understanding how pollinators are impacted by human‐altered landscapes, and how the resilience of nature's benefits to humans could be improved, is critical if we are to meet future societal needs.

## Author Contributions

**Jessica J. Williams:** conceptualization (lead), formal analysis (lead), methodology (lead), writing – original draft (lead), writing – review and editing (lead). **Tim Newbold:** formal analysis (supporting), funding acquisition (supporting), methodology (supporting), writing – review and editing (supporting). **Joseph Millard:** formal analysis (supporting), methodology (supporting), writing – review and editing (supporting). **Vivienne P. Groner:** formal analysis (supporting), methodology (supporting), writing – review and editing (supporting). **Richard G. Pearson:** formal analysis (supporting), funding acquisition (lead), methodology (supporting), writing – review and editing (supporting).

## Conflicts of Interest

The authors declare no conflicts of interest.

## Supporting information


Data S1.

## Data Availability

All data and code needed to replicate the analyses are available in a Figshare repository (https://doi.org/10.6084/m9.figshare.23545788.v1). The raw PREDICTS Project database can be downloaded from https://data.nhm.ac.uk/dataset/the‐2016‐release‐of‐the‐predicts‐database. The European Space Agency Climate Change Initiative land‐cover maps can be downloaded from https://catalogue.ceda.ac.uk/uuid/b382ebe6679d44b8b0e68ea4ef4b701c. For the datasets used to produce the species‐level ecosystem service contribution matrix, please see the original papers: Klein et al. (2007), DOI: 10.1098/rspb.2006.3721, and Millard et al. (2021), DOI: 10.1038/s41467‐021‐23228‐3 (and DOI: 10.5281/zenodo.7385950 for the complete list of pollinating species).

## Appendix A

**TABLE A1:** The number of assemblages from the PREDICTS Project database (Hudson et al. 2017) containing species within the contribution groups in each land‐use‐use‐intensity category within each model run. The contribution groups (low, medium, and high) indicate the contribution of those species towards the provision of crop pollination.

Land‐use type	Use intensity	Species richness model			Total abundance model		
		Low‐contribution group	Medium‐contribution group	High‐contribution group	Low‐contribution group	Medium‐contribution group	High‐contribution group
Primary vegetation	Minimal	1409	1082	895	1352	1043	847
	Light	1085	860	703	895	664	513
	Intense	389	363	199	355	329	165
Secondary vegetation	Minimal	826	568	360	758	500	292
	Light	289	201	188	277	199	186
	Intense	298	235	191	286	235	191
Plantation forest	Minimal	281	193	194	275	187	188
	Light	840	497	534	793	450	464
	Intense	163	83	98	152	73	81
Cropland	Minimal	307	321	300	307	321	300
	Light	134	396	535	133	395	534
	Intense	178	455	612	178	455	611
Pasture	Minimal	266	268	263	266	268	263
	Light	434	443	186	428	443	184
	Intense	52	99	108	46	99	101
Urban	Minimal	163	108	113	163	108	113
	Light	264	118	125	264	118	125
	Intense	94	39	39	94	39	39

**TABLE A2:** The selection of models tested within the backwards stepwise variable selection process. The fit of different models were compared using likelihood‐ratio tests. For the species richness and total abundance models, ΔAIC values are given in comparison with the best‐performing model (the model including the most complex fixed‐effects structure possible in each circumstance). The marginal *R* ^2^ value of each model is also provided. To explore the impact of contribution group and land‐use‐use‐intensity on species richness, we used generalised linear mixed‐effects models with a Poisson error distribution, with all models tested including 3 random intercept terms (study identity, spatial block, and site identity). To investigate the impact of contribution group, land‐use‐use‐intensity, and percentage of surrounding semi‐natural habitat (SNH) on total abundance, we used linear mixed‐effects models, with all models tested including study identity and spatial block as random intercept terms. See the main text for more information on the variables included in each model.

Model	ΔAIC	Marginal *R* ^2^
Fixed effects included		
**Species richness**		
Contribution group, land‐use‐use‐intensity, and the interaction between them	0	0.14
Contribution group and land‐use‐use‐intensity	14,241	0.07
Contribution group only	14,582	0.06
Land‐use‐use‐intensity only	26,382	0.01
None (null model)	26,723	0
**Total abundance**		
Contribution group, land‐use‐use‐intensity, and percentage of surrounding SNH, and the 2‐ and 3‐way interactions between them	0	0.10
Contribution group, land‐use‐use‐intensity, and percentage of surrounding SNH, and the 2‐way interactions between them	787	0.09
Contribution group, land‐use‐use‐intensity, and percentage of surrounding SNH	3202	0.05
Contribution group only	3303	0.05
Land‐use‐use‐intensity only	5146	0.01
Percentage of surrounding SNH only	5217	< 0.01
None (null model)	5230	0

**TABLE A3:** The coefficients, standard errors, and *p* values from the generalised linear mixed‐effects model with a Poisson error distribution investigating the impact of contribution group, land‐use type and land‐use intensity on species richness. Land‐use types included primary vegetation (PV), secondary vegetation (SV), plantation forest (PF), cropland (Cr), pasture (Pa), and urban (Ur). Land‐use intensities ranged from minimal, to light, to intense use. Species contribution groupings included those in the low‐, medium‐, and high‐contribution groups. Colons represent interaction terms. We applied a quasi‐likelihood analysis to adjust for overdispersion.

Term	Estimate	Standard error	*p*
(Intercept)	2.07	0.12	< 0.001
PV light use	0.32	0.04	< 0.001
PV intense use	0.43	0.05	< 0.001
SV minimal use	−0.32	0.04	< 0.001
SV light use	−0.04	0.05	0.428
SV intense use	−0.21	0.07	0.001
PF minimal use	0.11	0.06	0.088
PF light use	−0.21	0.05	< 0.001
PF intense use	−0.23	0.09	0.006
Cr minimal use	−0.37	0.07	< 0.001
Cr light use	−0.27	0.08	< 0.001
Cr intense use	−0.95	0.09	< 0.001
Pa minimal use	−0.35	0.06	< 0.001
Pa light use	−0.47	0.06	< 0.001
Pa intense use	−0.57	0.13	< 0.001
Ur minimal use	−0.03	0.08	0.747
Ur light use	−0.49	0.09	< 0.001
Ur intense use	−0.79	0.13	< 0.001
Medium‐contribution group	−0.85	0.03	< 0.001
High‐contribution group	−1.68	0.05	< 0.001
PV light use: Medium‐contribution group	−0.33	0.05	< 0.001
PV intense use: Medium‐contribution group	−0.91	0.07	< 0.001
SV minimal use: Medium‐contribution group	0.96	0.04	< 0.001
SV light use: Medium‐contribution group	0.36	0.05	< 0.001
SV intense use: Medium‐contribution group	0.51	0.07	< 0.001
PF minimal use: Medium‐contribution group	−0.64	0.09	< 0.001
PF light use: Medium‐contribution group	0.12	0.06	0.027
PF intense use: Medium‐contribution group	−0.33	0.11	0.003
Cr minimal use: Medium‐contribution group	−0.06	0.09	0.471
Cr light use: Medium‐contribution group	−0.87	0.11	< 0.001
Cr intense use: Medium‐contribution group	−0.16	0.12	0.177
Pa minimal use: Medium‐contribution group	0.30	0.06	< 0.001
Pa light use: Medium‐contribution group	1.07	0.06	< 0.001
Pa intense use: Medium‐contribution group	0.09	0.19	0.615
Ur minimal use: Medium‐contribution group	−0.56	0.10	< 0.001
Ur light use: Medium‐contribution group	0.10	0.11	0.362
Ur intense use: Medium‐contribution group	0.28	0.20	0.157
PV light use: High‐contribution group	−0.23	0.07	0.002
PV intense use: High‐contribution group	−0.91	0.15	< 0.001
SV minimal use: High‐contribution group	0.69	0.07	< 0.001
SV light use: High‐contribution group	−0.18	0.12	0.120
SV intense use: High‐contribution group	−0.14	0.14	0.295
PF minimal use: High‐contribution group	−0.05	0.11	0.669
PF light use: High‐contribution group	0.26	0.08	0.001
PF intense use: High‐contribution group	0.01	0.14	0.961
Cr minimal use: High‐contribution group	1.58	0.09	< 0.001
Cr light use: High‐contribution group	1.48	0.10	< 0.001
Cr intense use: High‐contribution group	2.45	0.11	< 0.001
Pa minimal use: High‐contribution group	0.98	0.09	< 0.001
Pa light use: High‐contribution group	0.83	0.11	< 0.001
Pa intense use: High‐contribution group	1.82	0.16	< 0.001
Ur minimal use: High‐contribution group	0.88	0.12	< 0.001
Ur light use: High‐contribution group	1.97	0.10	< 0.001
Ur intense use: High‐contribution group	1.97	0.16	< 0.001

**TABLE A4:** The coefficients and their *p* values from the linear mixed‐effects model investigating the impact of contribution group, land‐use type, land‐use intensity, and percentage of surrounding semi‐natural habitat (SNH) on total abundance. Land‐use types included primary vegetation (PV), secondary vegetation (SV), plantation forest (PF), cropland (Cr), pasture (Pa), and urban (Ur). Land‐use intensities ranged from minimal, to light, to intense use. Species contribution groupings included those in the low‐, medium‐, and high‐contribution groups. Colons represent interaction terms. We evaluated significance for coefficients using the Kenward‐Roger approximation for degrees of freedom (Luke 2017). Semi‐natural habitat was run as a first‐degree (i.e., linear) orthogonal polynomial in the model.

Term	Estimate	*p*
Intercept	3.77	< 0.001
PV light use	0.21	< 0.001
PV intense use	0.73	< 0.001
SV minimal use	−0.79	< 0.001
SV light use	−0.25	0.009
SV intense use	−0.42	< 0.001
PF minimal use	0.24	0.015
PF light use	−0.60	< 0.001
PF intense use	−0.36	0.004
Cr minimal use	−0.26	0.006
Cr light use	−0.53	< 0.001
Cr intense use	−1.06	< 0.001
Pa minimal use	0.17	0.071
Pa light use	−0.30	< 0.001
Pa intense use	−0.96	< 0.001
Ur minimal use	−0.19	0.246
Ur light use	−0.24	0.101
Ur intense use	−0.33	0.363
SNH	27.00	< 0.001
High‐contribution group	−1.51	< 0.001
Medium‐contribution group	−1.25	< 0.001
PV light use: High‐contribution group	−0.07	0.447
PV intense use: High‐contribution group	−0.93	< 0.001
SV minimal use: High‐contribution group	1.04	< 0.001
SV light use: High‐contribution group	0.03	0.845
SV intense use: High‐contribution group	−0.003	0.982
PF minimal use: High‐contribution group	−0.06	0.634
PF light use: High‐contribution group	0.22	0.017
PF intense use: High‐contribution group	0.72	< 0.001
Cr minimal use: High‐contribution group	1.29	< 0.001
Cr light use: High‐contribution group	1.59	< 0.001
Cr intense use: High‐contribution group	2.19	< 0.001
Pa minimal use: High‐contribution group	−0.39	0.004
Pa light use: High‐contribution group	0.28	0.023
Pa intense use: High‐contribution group	2.28	< 0.001
Ur minimal use: High‐contribution group	1.38	< 0.001
Ur light use: High‐contribution group	−0.53	0.021
Ur intense use: High‐contribution group	−0.59	0.460
PV light use: Medium‐contribution group	0.02	0.765
PV intense use: Medium‐contribution group	−0.55	< 0.001
SV minimal use: Medium‐contribution group	1.61	< 0.001
SV light use: Medium‐contribution group	0.98	< 0.001
SV intense use: Medium‐contribution group	1.01	< 0.001
PF minimal use: Medium‐contribution group	−0.22	0.088
PF light use: Medium‐contribution group	0.66	< 0.001
PF intense use: Medium‐contribution group	0.48	0.008
Cr minimal use: Medium‐contribution group	0.11	0.357
Cr light use: Medium‐contribution group	−0.21	0.184
Cr intense use: Medium‐contribution group	0.65	< 0.001
Pa minimal use: Medium‐contribution group	−0.02	0.896
Pa light use: Medium‐contribution group	1.09	< 0.001
Pa intense use: Medium‐contribution group	1.22	< 0.001
Ur minimal use: Medium‐contribution group	0.39	0.120
Ur light use: Medium‐contribution group	1.33	< 0.001
Ur intense use: Medium‐contribution group	1.07	0.182
PV light use:SNH	−6.40	0.434
PV intense use:SNH	−23.46	0.116
SV minimal use:SNH	67.67	< 0.001
SV light use:SNH	−69.49	< 0.001
SV intense use:SNH	71.53	< 0.001
PF minimal use:SNH	−43.45	< 0.001
PF light use:SNH	−6.83	0.445
PF intense use:SNH	−27.64	0.126
Cr minimal use:SNH	9.23	0.399
Cr light use:SNH	34.98	0.014
Cr intense use:SNH	31.40	0.016
Pa minimal use:SNH	−26.68	0.065
Pa light use:SNH	−88.96	< 0.001
Pa intense use:SNH	−115.27	< 0.001
Ur minimal use:SNH	−62.35	0.001
Ur light use:SNH	16.48	0.241
Ur intense use:SNH	47.95	0.183
SNH:High‐contribution group	−33.09	< 0.001
SNH:Medium‐contribution group	−60.42	< 0.001
PV light use:SNH:High‐contribution group	−56.45	< 0.001
PV intense use:SNH:High‐contribution group	61.44	0.003
SV minimal use:SNH:High‐contribution group	−86.26	< 0.001
SV light use:SNH:High‐contribution group	155.01	< 0.001
SV intense use:SNH:High‐contribution group	−58.19	0.021
PF minimal use:SNH:High‐contribution group	64.81	< 0.001
PF light use:SNH:High‐contribution group	119.27	< 0.001
PF intense use:SNH:High‐contribution group	142.86	< 0.001
Cr minimal use:SNH:High‐contribution group	−43.03	0.006
Cr light use:SNH:High‐contribution group	−69.01	< 0.001
Cr intense use:SNH:High‐contribution group	−79.81	< 0.001
Pa minimal use:SNH:High‐contribution group	97.26	< 0.001
Pa light use:SNH:High‐contribution group	98.72	< 0.001
Pa intense use:SNH:High‐contribution group	111.15	< 0.001
Ur minimal use:SNH:High‐contribution group	136.65	< 0.001
Ur light use:SNH:High‐contribution group	−219.93	< 0.001
Ur intense use:SNH:High‐contribution group	−255.50	0.001
PV light use:SNH:Medium‐contribution group	63.46	< 0.001
PV intense use:SNH:Medium‐contribution group	15.42	0.422
SV minimal use:SNH:Medium‐contribution group	−72.85	< 0.001
SV light use:SNH:Medium‐contribution group	89.94	< 0.001
SV intense use:SNH:Medium‐contribution group	−108.57	< 0.001
PF minimal use:SNH:Medium‐contribution group	68.28	< 0.001
PF light use:SNH:Medium‐contribution group	67.39	< 0.001
PF intense use:SNH:Medium‐contribution group	42.97	0.153
Cr minimal use:SNH:Medium‐contribution group	33.31	0.024
Cr light use:SNH:Medium‐contribution group	−9.42	0.600
Cr intense use:SNH:Medium‐contribution group	34.73	0.035
Pa minimal use:SNH:Medium‐contribution group	5.37	0.775
Pa light use:SNH:Medium‐contribution group	122.18	< 0.001
Pa intense use:SNH:Medium‐contribution group	204.25	< 0.001
Ur minimal use:SNH:Medium‐contribution group	95.53	0.002
Ur light use:SNH:Medium‐contribution group	75.40	0.003
Ur intense use:SNH:Medium‐contribution group	43.84	0.583
