# Supplementary material for: Important Crop Pollinators Respond Less Negatively to Anthropogenic Land Use Than Other Animals
Source: Ecol Evol. 2024 Oct 30;14(11):e70486. doi: 10.1002/ece3.70486 (PMC11522614; doi:10.1002/ece3.70486)
Supplement: Supplementary file 1 — Data S1. [file ECE3-14-e70486-s001.zip › ece370486-sup-0001-Supinfo.docx]

Important crop pollinators respond less negatively to anthropogenic land use than other animals

Supporting Information

Corresponding author, [jessica.williams.16@ucl.ac.uk](mailto:jessica.williams.16@ucl.ac.uk)

Description of the databases

*PREDICTS Project database*

Table S1: Classifications of the land-use types and intensities (table adapted from Hudson et al. 2014).

| **Level 1 Land Use** | **Predominant Land Use** | **Minimal use** | **Light use** | **Intense use** |
| --- | --- | --- | --- | --- |
| No evidence of prior destruction of the vegetation | Primary Vegetation | Any disturbances identified are very minor (e.g., a trail or path) or very limited in the scope of their effect (e.g., hunting of a particular species of limited ecological importance). | One or more disturbances of moderate intensity (e.g., selective logging) or breadth of impact (e.g., bushmeat extraction), which are not severe enough to markedly change the nature of the ecosystem. Primary sites in suburban settings are at least Light use. | One or more disturbances that is severe enough to markedly change the nature of the ecosystem; this includes clear-felling of part of the site too recently for much recovery to have occurred. Primary sites in fully urban settings should be classed as Intense use. |
| Recovering after destruction of the vegetation | Secondary Vegetation | As for Primary Vegetation-Minimal use | As for Primary Vegetation-Light use | As for Primary Vegetation-Intense use |
| Human use (agricultural) | Plantation forest | Extensively managed or mixed timber, fruit/coffee, oil-palm or rubber plantations in which native understorey and/or other native tree species are tolerated, which are not treated with pesticide or fertiliser, and which have not been recently (< 20 years) clear-felled. | Monoculture fruit/coffee/rubber plantations with limited pesticide input, or mixed species plantations with significant inputs. Monoculture timber plantations of mixed age with no recent (< 20 years) clear-felling. Monoculture oil-palm plantations with no recent (< 20 years) clear-felling. | Monoculture fruit/coffee/rubber plantations with significant pesticide input.  Monoculture timber plantations with similarly aged trees or timber/oil-palm plantations with extensive recent (< 20 years) clear-felling. |
| Human use (agricultural) | Cropland | Low-intensity farms, typically with small fields, mixed crops, crop rotation, little or no inorganic fertiliser Human use (agricultural)use, little or no pesticide use, little or no ploughing, little or no irrigation, little or no mechanisation. | Medium intensity farming, typically showing some but not many of the following: large fields, annual ploughing, inorganic fertiliser application, pesticide application, irrigation, no crop rotation, mechanisation, monoculture crop. Organic farms in developed countries often fall within this category, as may high-intensity farming in developing countries. | High-intensity monoculture farming, typically showing many of the following features: large fields, annual ploughing, inorganic fertiliser application, pesticide application, irrigation, mechanisation, no crop rotation. |
| Human use (agricultural) | Pasture | Pasture with minimal input of fertiliser and pesticide, and with low stock density (*not* high enough to cause significant disturbance or to stop regeneration of vegetation). | Pasture either with significant input of fertiliser or pesticide, or with high stock density (high enough to cause significant disturbance or to stop regeneration of vegetation). | Pasture with significant input of fertiliser or pesticide, *and* with high stock density (high enough to cause significant disturbance or to stop regeneration of vegetation). |
| Human use (urban) | Urban | Extensive managed green spaces; villages. | Suburban (e.g. gardens), or small managed or unmanaged green spaces in cities. | Fully urban with no significant green spaces. |

*European Space Agency Climate Change Initiative land-cover maps*

Table S2: The 37 land-cover categories classified by the European Space Agency Climate Change Initiative (ESA CCI; ESA Land Cover CCI project team, Defourny, 2019). The ESA CCI land-cover maps use a hierarchical classification – level 1 (the ‘global’ legend) is defined using the ten value codes (i.e., 10, 20, 30…) and level 2 (the ‘regional’ legend, which is a subcategory of level 1) is defined using the non-ten values (i.e., 11, 12, 13…). Following Williams, Freeman, Spooner, & Newbold, (2022), the categories were grouped to form a semi-natural habitat (SNH) category in order to calculate the percentage of surrounding SNH within a 1-km radius of each assemblage. We accounted for the maximum percentage cover of a specific land cover (detailed in the ESA’s land-cover categories). This table is adapted from Williams et al. (2022).

| **Land cover categories used in the ESA CCI land-cover maps^▲^** | | | **Included as SNH? (Y/N)** | **Maximum percentage cover of SNH (%)** |
| --- | --- | --- | --- | --- |
| **Legend values** | | **Labels** |  |  |
| Level 1   - Level 2 | |  |  |  |
| 10 |  | Rainfed cropland | N | 0 |
| - 11 | | - Herbaceous cover | N | 0 |
| - 12 | | - Tree or shrub cover | N | 0 |
| 20 |  | Irrigated cropland | N | 0 |
| 30 |  | Mosaic cropland (>50%) / natural vegetation (tree, shrub, herbaceous cover) (<50%) | N | 0 |
| 40 |  | Mosaic natural vegetation (tree, shrub, herbaceous cover) (>50%) / cropland (<50%) | N | 0 |
| 100^+^ |  | Mosaic tree and shrub (>50%) / herbaceous cover (<50%) | N | 0 |
| 50 |  | Tree cover, broadleaved, evergreen, closed to open (>15%) | Y | 100 |
| 60 |  | Tree cover, broadleaved, deciduous, closed to open (>15%) | Y | 100 |
| - 61 | | - Tree cover, broadleaved, deciduous, closed (>40%) | Y | 100 |
| - 62 | | - Tree cover, broadleaved, deciduous, open (15-40%) | Y | 40 |
| 70 |  | Tree cover, needleleaved, evergreen, closed to open (>15%) | Y | 100 |
| - 71 | | - Tree cover, needleleaved, evergreen, closed (>40%) | Y | 100 |
| - 72 | | - Tree cover, needleleaved, evergreen, open (15-40%) | Y | 40 |
| 80 |  | Tree cover needleleaved, deciduous, closed to open (>15%) | Y | 100 |
| - 81 | | - Tree cover, needleleaved, deciduous, closed (>40%) | Y | 100 |
| - 82 | | - Tree cover, needleleaved, deciduous, open (15-40%) | Y | 40 |
| 90 |  | Tree cover, mixed leaf type (broad leaved and needleleaved) | Y | 100 |
| 160 |  | Tree cover, flooded, fresh or brackish water | Y | 100 |
| 170 |  | Tree cover, flooded, saline water | Y | 100 |
| 110 |  | Mosaic herbaceous cover (>50%) / tree and shrub (<50%) | Y | 50 |
| 130 |  | Grassland | Y | 100 |
| 180 |  | Shrub or herbaceous cover, flooded, fresh-saline or brackish water | Y | 100 |
| 190 |  | Urban | N | 0 |
| 120 |  | Shrubland | Y | 100 |
| - 121 | | - Evergreen shrubland | Y | 100 |
| - 122 | | - Deciduous shrubland | Y | 100 |
| 140 |  | Lichens and mosses | N | 0 |
| 150 |  | Sparse vegetation (tree, shrub, herbaceous cover) | N | 0 |
| - 152 | | - Sparse shrub (<15%) | N | 0 |
| - 153 | | - Sparse herbaceous cover (<15%) | N | 0 |
| 200 |  | Bare areas | N | 0 |
| - 201 | | - Consolidated bare areas | N | 0 |
| - 202 | | - Unconsolidated bare areas | N | 0 |
| 210 |  | Water | N | 0 |
| 220 |  | Permanent snow and ice | N | 0 |

^+^ This was classed as agriculture (i.e., not semi-natural habitat) due to communications with members of the Sentinel (Social and Environmental Trade-Offs in African Agriculture) Project (www.sentinel-gcrf.org), who have found that this land-cover category was commonly cropland with sparse trees.

^▲^ The 37^th^ category is a No Data category.

*Pollinator dataset constructed by Millard et al. (2021)*

Millard et al. (2021) used an automatic text-analysis method and manual inspection to create a list of likely pollinating genera (for any flowering plant). Within this dataset, the authors produced a four-level confidence ranking, which included (from highest to lowest confidence) (a)“evidence of non-destructive/nonpredatory flower visitations”, (b) “evidence of nectar/pollen feeding”, (c) “evidence of pollen carrying” and (d) “experimental evidence confirming pollination” (Millard et al., 2021). They also carried out searches within higher-level taxonomic groups to extrapolate across genera if there was sufficient evidence. In addition, this pollinator dataset was assessed by experts (Millard et al., 2021).

We used the confidence rankings within this dataset, and whether the pollinator evidence had been extrapolated or not, to assign species importance and certainty levels, respectively, in our species-level ecosystem service contribution matrix.

*Global dataset on crops and their pollinators constructed by Klein et al. (2007)*

Klein et al. (2007) produced a dataset containing 107 crops from around the world that produce seeds or fruits for direct use as food by humans (FAOSTAT 2005), and, by completing an extensive literature review, the level of dependence of each of these crops on animal-mediated pollination. Within this dataset, they list the animal groups or species that are known to be “important flower visitors or pollinators” for each crop (Klein et al., 2007). They classify *true pollinators* or *primary pollinators* as “species for which at least 80% of their single flower visits results in a fruit (Klein, Steffan-Dewenter, & Tscharntke, 2003a, 2003b) or species that improve the fruit and seed quality and quantity when abundant as compared with the level when all flower visitors are excluded” (Klein et al., 2007). Then, they state the “magnitude of improvement in production and quality when [the crop is] pollinated by animals”, i.e., the level of dependence of that crop on animal-mediated pollination. For this, they had 6 categories:

1. Essential = the crop requires animal pollination, with at least a 90% reduction in production without flower visitors
2. High = there is a 40 – <90% reduction in production without flower visitors
3. Modest = there is a 10 – <40% reduction in production without flower visitors
4. Little = there is 0 – <10% reduction in production without flower visitors
5. No reduction = no reduction in production without flower visitors
6. Unknown = “no literature was available to adequately review the breeding systems or draw conclusions about pollinator dependence”

We used these categories, and whether a genera or species was listed as a pollinator or flower visitor, to assign species importance and certainty levels in our species-level ecosystem service contribution matrix.

Further information the results

Table S3: The number of species within the Classes contained in the final dataset across each contribution group (low, medium, or high; where contribution indicates the species’ contribution towards provision of crop pollination).

| Classes contained within the dataset | Contribution group | | |
| --- | --- | --- | --- |
|  | Low | Medium | High |
| Aves | 1559 | 315 | 135 |
| Insecta | 3883 | 2799 | 599 |
| Mammalia | 144 | 10 | 34 |
| Reptilia | 293 | 3 | 2 |

Table S4: The number of species within each Order contained in each contribution group (low, medium, or high) in the final dataset, where contribution indicates the species’ contribution towards the provision of crop pollination.

*See Table_S4.csv*

Table S5: The names of the species included in each contribution group in the final dataset. The contribution groups indicate the contribution of a species to the provision of crop pollination – (1) low, (2) medium, and (3) high.

*See Table_S5.csv*

Table S6: The number of studies within the PREDICTS Project database that included species in each contribution group in the final dataset, where contribution indicates the contribution of the species towards the provision of crop pollination.

| Studies including species from… | | Number of studies |
| --- | --- | --- |
|  | The low-contribution group only | 109 |
|  | The medium-contribution group only | 11 |
|  | The high-contribution group only | 27 |
|  | The low and medium-contribution group | 28 |
|  | The low and high-contribution group | 8 |
|  | The medium and high-contribution group | 26 |
|  | All contribution groups | 117 |

Table S7: The number of assemblages at tropical latitudes from the PREDICTS Project database (Hudson et al., 2017) across each land-use-use-intensity category that were included in the final dataset. The table is split by the data available for species in that assemblage (species richness and/or abundance data). The contribution groups (low, medium, and high) indicate the contribution of the species towards the provision of crop pollination.

| **Land-use type** | **Use intensity** | **Data available** | | | | | |
| --- | --- | --- | --- | --- | --- | --- | --- |
|  |  | Species richness | | | Total abundance | | |
|  |  | Low-contribution group | Medium-contribution group | High-contribution group | Low-contribution group | Medium-contribution group | High-contribution group |
| Primary vegetation | Minimal | 648 | 424 | 396 | 592 | 385 | 348 |
|  | Light | 525 | 322 | 299 | 489 | 280 | 263 |
|  | Intense | 248 | 224 | 126 | 240 | 216 | 118 |
| Secondary vegetation | Minimal | 346 | 236 | 190 | 344 | 234 | 188 |
|  | Light | 191 | 141 | 140 | 181 | 141 | 140 |
|  | Intense | 163 | 120 | 62 | 151 | 120 | 62 |
| Plantation forest | Minimal | 181 | 103 | 104 | 175 | 97 | 98 |
|  | Light | 779 | 458 | 495 | 742 | 421 | 435 |
|  | Intense | 58 | 25 | 40 | 57 | 25 | 33 |
| Cropland | Minimal | 141 | 93 | 71 | 141 | 93 | 71 |
|  | Light | 104 | 86 | 73 | 103 | 85 | 72 |
|  | Intense | 87 | 78 | 66 | 87 | 78 | 66 |
| Pasture | Minimal | 44 | 19 | 24 | 44 | 19 | 24 |
|  | Light | 196 | 84 | 51 | 190 | 84 | 51 |
|  | Intense | 15 | 9 | 1 | 9 | 9 | 1 |
| Urban | Minimal | 11 | 8 | 0 | 11 | 8 | 0 |
|  | Light | 45 | 43 | 43 | 45 | 43 | 43 |
|  | Intense | 4 | 2 | 2 | 4 | 2 | 2 |

Table S8: The number of assemblages at temperate latitudes from the PREDICTS Project database (Hudson et al., 2017) across each land-use-use-intensity category that were included in the final dataset. The table is split by the data available for species in that assemblage (species richness and/or abundance data). The contribution groups (low, medium, and high) indicate the contribution of the species towards the provision of crop pollination.

| **Land-use type** | **Use intensity** | **Data available** | | | | | |
| --- | --- | --- | --- | --- | --- | --- | --- |
|  |  | Species richness | | | Total abundance | | |
|  |  | Low-contribution group | Medium-contribution group | High-contribution group | Low-contribution group | Medium-contribution group | High-contribution group |
| Primary vegetation | Minimal | 761 | 658 | 499 | 761 | 658 | 499 |
|  | Light | 560 | 538 | 404 | 406 | 384 | 250 |
|  | Intense | 141 | 139 | 73 | 115 | 113 | 47 |
| Secondary vegetation | Minimal | 480 | 332 | 170 | 414 | 266 | 104 |
|  | Light | 98 | 60 | 48 | 96 | 58 | 46 |
|  | Intense | 135 | 115 | 129 | 135 | 115 | 129 |
| Plantation forest | Minimal | 100 | 90 | 90 | 100 | 90 | 90 |
|  | Light | 61 | 39 | 39 | 51 | 29 | 29 |
|  | Intense | 105 | 58 | 58 | 95 | 48 | 48 |
| Cropland | Minimal | 166 | 228 | 229 | 166 | 228 | 229 |
|  | Light | 30 | 310 | 462 | 30 | 310 | 462 |
|  | Intense | 91 | 388 | 546 | 91 | 377 | 545 |
| Pasture | Minimal | 222 | 249 | 239 | 222 | 249 | 239 |
|  | Light | 238 | 359 | 135 | 238 | 359 | 133 |
|  | Intense | 37 | 90 | 107 | 37 | 90 | 100 |
| Urban | Minimal | 152 | 100 | 113 | 152 | 100 | 113 |
|  | Light | 219 | 75 | 82 | 219 | 75 | 82 |
|  | Intense | 90 | 37 | 37 | 90 | 37 | 37 |

Table S9: Summary statistics for the percentage of semi-natural habitat surrounding each PREDICTS Project site included in our analysis, shown across the whole dataset, and then split by geographic realm (tropical or temperate).

| Statistic | All Sites | Temperate Sites | Tropical Sites |
| --- | --- | --- | --- |
| Minimum | 0 | 0 | 0 |
| Maximum | 100 | 100 | 100 |
| Median | 73.26 | 55.26 | 88.00 |
| Mean | 60.20 | 52.10 | 71.25 |
| Standard deviation | 37.99 | 38.57 | 34.22 |

Table S10: Marginal and conditional R^2^ values for each model run.

| Model | Conditional R^2^ | Marginal R^2^ |
| --- | --- | --- |
| Species richness | 0.70 | 0.14 |
| Total abundance | 0.77 | 0.10 |

Sensitivity tests

1. *Chao1-estimated species richness*





Figure S1: The difference in species richness (accounting for species’ abundance, using Chao1-estimated species richness) in assemblages across different land-use types and land-use intensities, relative to that in minimally used primary vegetation. Assemblages have been split into three groups: (a) species in the low-contribution group; (b) species in the medium-contribution group; and (c) species in the high-contribution group. Colours represent land-use type: primary vegetation (PV; light green), secondary vegetation (SV; dark green), plantation (purple), cropland (yellow), pasture (orange), and urban (red). Error bars represent ± 1 standard error. The down arrows for the high-contribution group in intense use primary vegetation, secondary vegetation, and plantation, and light use secondary vegetation represent predictions by the model that there may be no species from the high-contribution group in these land uses. Note the difference in y-axis limits between the plots.

1. *Zero-inflated negative binomial mixed models*





Figure S2: The difference in total abundance of species in assemblages across different land-use types, land-use intensities, and with different amounts of surrounding semi-natural habitat (SNH), relative to assemblages in minimally used primary vegetation surrounded by a high percentage (91.4%) of SNH, produced using a zero-inflated negative binomial mixed model. Assemblages have been split into three groups: (a) species in the low-contribution group; (b) species in the medium-contribution group; and (c) species in the high-contribution group. Open shapes represent those assemblages with a low percentage of surrounding SNH (37.5%) and filled shapes represent those assemblages with a high percentage of surrounding SNH (91.4%) – to enable comparison to the plots in the main text. Colours represent land-use type: primary vegetation (PV; light green), secondary vegetation (SV; dark green), plantation (purple), cropland (yellow), pasture (orange), and urban (red). Error bars represent ± 1 standard error. The down arrows for the medium-contribution group in light use cropland represent predictions by the model that there may be no species (abundance = 0) from this contribution group in these land uses.

1. *Spatial autocorrelation*

We ran an ‘overall’ species richness and total abundance model (i.e., by removing the contribution grouping and including the total number of species or abundance of individuals at a site, respectively). Using Moran’s I tests, we observed in the residuals of the two models that slightly more than 5% of the studies returned *p* < 0.05 (table S11).

Table S11: Spatial autocorrelation in the model residuals; a Moran’s I test was applied to the residuals to the two ‘overall’ models for each individual underlying study separately, with the percentage of studies that had *p* < 0.05 reported below. In the absence of spatial autocorrelation, we could expect by chance that 5% of studies would return a *p* value of < 0.05.

| Model | Percentage of studies for which *p* < 0.05 | The distribution of *p* values (the red dotted line represents a *p* value of 0.05) |
| --- | --- | --- |
| Species richness | 8.29 |  |
| Total abundance | 5.80 |  |

1. *Outliers*





Figure S3: The difference in total abundance of species in assemblages across different land-use types, land-use intensities, and with different amounts of surrounding semi-natural habitat (SNH), relative to assemblages in minimally used primary vegetation surrounded by a high percentage (91.4%) of SNH, after removing extreme values of total abundance. Assemblages have been split into three groups: (a) species in the low-contribution group; (b) species in the medium-contribution group; and (c) species in the high-contribution group. Open shapes represent those assemblages with a low percentage of surrounding SNH (37.5%) and filled shapes represent those assemblages with a high percentage of surrounding SNH (91.4%)– these values were the 66th and 33rd percentile of sampled sites before outliers were removed, respectively (to enable comparison to the main text). Colours represent land-use type: primary vegetation (PV; light green), secondary vegetation (SV; dark green), plantation (purple), cropland (yellow), pasture (orange), and urban (red). Error bars represent ± 1 standard error.

1. *Cross validation tests*





Figure S4: The difference in species richness in assemblages across different land-use types and land-use intensities, relative to that in minimally used primary vegetation. Values are shown for the original model, and models run using the lower (2.5^th^ percentile) and upper (97.5^th^ percentile) confidence intervals for coefficients when each study in our dataset was removed one at a time and the models rerun. Assemblages have been split into three groups: (a) species in the low-contribution group; (b) species in the medium-contribution group; and (c) species in the high-contribution group. Colours represent land-use type: primary vegetation (PV; light green), secondary vegetation (SV; dark green), plantation (purple), cropland (yellow), pasture (orange), and urban (red). Note the difference in y-axis limits between the plots.





Figure S5: The difference in total abundance of species in assemblages across different land-use types, land-use intensities, and with different amounts of surrounding semi-natural habitat (SNH), relative to assemblages in minimally used primary vegetation surrounded by a high percentage (91.4%) of SNH. Values are shown for the original model, and models run using the lower (2.5^th^ percentile) and upper (97.5^th^ percentile) confidence intervals for coefficients when each study in our dataset was removed one at a time and the models rerun. Assemblages have been split into three groups: (a) species in the low-contribution group; (b) species in the medium-contribution group; and (c) species in the high-contribution group. Open shapes represent those assemblages with a low percentage of surrounding SNH (37.5%) and filled shapes represent those assemblages with a high percentage of surrounding SNH (91.4%)– these values were the 66th and 33rd percentile, respectively, across sampled sites (to enable comparison to the main text). Colours represent land-use type: primary vegetation (PV; light green), secondary vegetation (SV; dark green), plantation (purple), cropland (yellow), pasture (orange), and urban (red).

1. *Contribution groupings based on importance only*

We ran the models as described in the main text, but grouped species by their importance classification only, and did not down-weight a species’ contribution to pollination by the level of certainty of the evidence for the species’ importance. As such the low-contribution group included species whose importance for crop pollination was negligible, the medium-contribution group included those species that were of low importance for crop pollination, and the high-contribution group included species whose importance to crop pollination was classified as medium to very high (fig. 1).





Figure S6: The difference in species richness in assemblages across different land-use types and land-use intensities, relative to that in minimally used primary vegetation, when contribution groupings were based on species’ importance for pollination. Assemblages have been split into three groups: (a) species in the low-contribution group; (b) species in the medium-contribution group; and (c) species in the high-contribution group. Colours represent land-use type: primary vegetation (PV; light green), secondary vegetation (SV; dark green), plantation (purple), cropland (yellow), pasture (orange), and urban (red). Error bars represent ± 1 standard error. The down arrow for the high-contribution group in intense use primary and secondary vegetation represent predictions by the model that there may be no species from the high-contribution group in these land uses. The down arrow for the high-contribution group in intense use primary and secondary vegetation represent predictions by the model that there may be no species from the high-contribution group found in these land uses. Note the difference in y-axis limits between the plots.





Figure S7: The difference in total abundance of species in assemblages across different land-use types, land-use intensities, and with different amounts of surrounding semi-natural habitat (SNH), relative to assemblages in minimally used primary vegetation surrounded by a high percentage (91.4%) of SNH. Assemblages have been split into three groups: (a) species in the low-contribution group; (b) species in the medium-contribution group; and (c) species in the high-contribution group, with contribution groupings based on species’ importance for pollination. Open shapes represent those assemblages with a low percentage of surrounding SNH (37.5%) and filled shapes represent those assemblages with a high percentage of surrounding SNH (91.4%) – we chose to present these values as they were the 33^rd^ and 66^th^ percentile, respectively, across sampled sites. Colours represent land-use type: primary vegetation (PV; light green), secondary vegetation (SV; dark green), plantation (purple), cropland (yellow), pasture (orange), and urban (red). Error bars represent ± 1 standard error; the upper values of the error bars for intense use urban sites surrounded by a high percentage of SNH in plot (b) was 1269% and for minimal use urban sites surrounded by a high percentage of SNH in plot (c) was 968%.

7*. Including the percentage of SNH within a 10- or 50-km radius*





Figure S8: The difference in total abundance of species in assemblages across different land-use types, land-use intensities, and with different amounts of surrounding semi-natural habitat (SNH) within a 10 km radius, relative to assemblages in minimally used primary vegetation surrounded by a high percentage (91.4%) of SNH. Assemblages have been split into three groups: (a) species in the low-contribution group; (b) species in the medium-contribution group; and (c) species in the high-contribution group. Open shapes represent those assemblages with a low percentage of surrounding SNH (37.5%) and filled shapes represent those assemblages with a high percentage of surrounding SNH (91.4%) within a 10 km radius – to enable comparison to the plots in the main text. Colours represent land-use type: primary vegetation (PV; light green), secondary vegetation (SV; dark green), plantation (purple), cropland (yellow), pasture (orange), and urban (red). Error bars represent ± 1 standard error; the total abundance difference and upper value of the error bars for the medium-contribution group in light use urban sites surrounded by a high percentage of SNH were 868% and 1437%, respectively (plot (b)). This is likely due to a very small number of light use urban areas in the data having a high (>90%) percentage of SNH in the surrounding 10 km radius (n = 9).





Figure S9: The difference in total abundance of species in assemblages across different land-use types, land-use intensities, and with different amounts of surrounding semi-natural habitat (SNH) within a 50 km radius, relative to assemblages in minimally used primary vegetation surrounded by a high percentage (91.4%) of SNH. Assemblages have been split into three groups: (a) species in the low-contribution group; (b) species in the medium-contribution group; and (c) species in the high-contribution group. Open shapes represent those assemblages with a low percentage of surrounding SNH (37.5%) and filled shapes represent those assemblages with a high percentage of surrounding SNH (91.4%) within a 50 km radius – to enable comparison to the plots in the main text. Colours represent land-use type: primary vegetation (PV; light green), secondary vegetation (SV; dark green), plantation (purple), cropland (yellow), pasture (orange), and urban (red). Error bars represent ± 1 standard error. The upper value of the error bars for the medium-contribution group in the intense use pasture and urban sites surrounded by a high percentage of SNH were 1173% and 1403%, respectively (plot (b)). The total abundance difference and lower and upper values of the error bars for the medium-contribution group in light use urban sites surrounded by a high percentage of SNH were 5552%, 2601% and 11707%, respectively (plot (b)). This is likely due to a very small number of light use urban areas in the data having a high (>90%) percentage of SNH in the surrounding 50 km radius (n = 2).

8*. Including the percentage of SNH using land-cover data from 2002 and 2008*





Figure S10: The difference in total abundance of species in assemblages across different land-use types, land-use intensities, and with different amounts of surrounding semi-natural habitat (SNH) within a 1 km radius (based on land-cover data from 2002), relative to assemblages in minimally used primary vegetation surrounded by a high percentage (91.4%) of SNH. Assemblages have been split into three groups: (a) species in the low-contribution group; (b) species in the medium-contribution group; and (c) species in the high-contribution group. Open shapes represent those assemblages with a low percentage of surrounding SNH (37.5%) and filled shapes represent those assemblages with a high percentage of surrounding SNH (91.4%) – to enable comparison to the plots in the main text. Colours represent land-use type: primary vegetation (PV; light green), secondary vegetation (SV; dark green), plantation (purple), cropland (yellow), pasture (orange), and urban (red). Error bars represent ± 1 standard error. The upper value of the error bar for the medium-contribution group in the intense use urban site surrounded by a high percentage of SNH was 1288% (plot (b)).





Figure S11: The difference in total abundance of species in assemblages across different land-use types, land-use intensities, and with different amounts of surrounding semi-natural habitat (SNH) within a 1 km radius (based on land-cover data from 2008), relative to assemblages in minimally used primary vegetation surrounded by a high percentage (91.4%) of SNH. Assemblages have been split into three groups: (a) species in the low-contribution group; (b) species in the medium-contribution group; and (c) species in the high-contribution group. Open shapes represent those assemblages with a low percentage of surrounding SNH (37.5%) and filled shapes represent those assemblages with a high percentage of surrounding SNH (91.4%)– to enable comparison to the plots in the main text. Colours represent land-use type: primary vegetation (PV; light green), secondary vegetation (SV; dark green), plantation (purple), cropland (yellow), pasture (orange), and urban (red). Error bars represent ± 1 standard error. The upper value of the error bars for the medium-contribution group in the intense use urban site surrounded by a high percentage of SNH was 1176% (plot (b)).

References

ESA Land Cover CCI project team, Defourny, P. (2019). ESA Land Cover Climate Change Initiative (Land_Cover_cci): Global Land Cover Maps, Version 2.0.7. Centre for Environmental Data Analysis, downloaded on 28 January 2020. https://catalogue.ceda.ac.uk/uuid/b382ebe6679d44b8b0e68ea4ef4b701cFAOSTAT data, 2005. Data available at <http://faostat.fao.org>; Agricultural data/Agricultural production/Crops primary.

Hudson, L. N., Newbold, T., Contu, S., Hill, S. L. L., Lysenko, I., De Palma, A., … Purvis, A. (2014). The PREDICTS database: A global database of how local terrestrial biodiversity responds to human impacts. Ecology and Evolution, 4(24), 4701–4735. https://doi.org/10.1002/ece3.1303

Hudson, L. N., Newbold, T., Contu, S., Hill, S. L. L., Lysenko, I., De Palma, A., … Purvis, A. (2017). The database of the PREDICTS (Projecting Responses of Ecological Diversity In Changing Terrestrial Systems) project. Ecology and Evolution, 7(1), 145–188. https://doi.org/10.1002/ece3.2579

Klein, A. M., Steffan-Dewenter, I., & Tscharntke, T. (2003a). Fruit set of highland coffee increases with the diversity of pollinating bees. Proceedings of the Royal Society B: Biological Sciences, 270(1518), 955–961. https://doi.org/10.1098/rspb.2002.2306

Klein, A. M., Steffan-Dewenter, I., & Tscharntke, T. (2003b). Pollination of Coffea canephora in relation to local and regional agroforestry management. Journal of Applied Ecology, 40(5), 837–845. https://doi.org/10.1046/j.1365-2664.2003.00847.x

Klein, A. M., Vaissière, B. E., Cane, J. H., Steffan-Dewenter, I., Cunningham, S. A., Kremen, C., & Tscharntke, T. (2007). Importance of pollinators in changing landscapes for world crops. Proceedings of the Royal Society B: Biological Sciences, 274(1608), 303–313. <https://doi.org/10.1098/rspb.2006.3721>

Millard, J., Outhwaite, C. L., Kinnersley, R., Freeman, R., Gregory, R. D., Adedoja, O., … Newbold, T. (2021). Global effects of land-use intensity on local pollinator biodiversity. Nature Communications, 12(1), 1–11. https://doi.org/10.1038/s41467-021-23228-3

Williams, J. J., Freeman, R., Spooner, F., & Newbold, T. (2022). Vertebrate population trends are influenced by interactions between land use, climatic position, habitat loss and climate change. Global Change Biology, 28(3), 797–815. https://doi.org/10.1111/gcb.15978
